# Relation between functional magnetic resonance imaging (fMRI) and single neuron, local field potential (LFP) and electrocorticography (ECoG) activity in human cortex

**DOI:** 10.3389/fnhum.2013.00034

**Published:** 2013-02-20

**Authors:** George A. Ojemann, Nick F. Ramsey, Jeffrey Ojemann

**Affiliations:** ^1^Neurological Surgery, University of WashingtonSeattle, WA, USA; ^2^Neurology and Neurosurgery, University Medical CenterUtrecht, Netherlands; ^3^Seattle Children's HospitalSeattle, WA, USA

**Keywords:** fMRI BOLD, electrocorticogram, single neurons, local field potentials, human cortex

## Abstract

The relation between changes in the blood oxygen dependent metabolic changes imaged by functional magnetic resonance imaging (fMRI) and neural events directly recorded from human cortex from single neurons, local field potentials (LFPs) and electrocorticogram (ECoG) is critically reviewed, based on the published literature including findings from the authors' laboratories. All these data are from special populations, usually patients with medically refractory epilepsy, as this provides the major opportunity for direct cortical neuronal recording in humans. For LFP and ECoG changes are often sought in different frequency bands, for single neurons in frequency of action potentials. Most fMRI studies address issues of functional localization. The relation of those findings to localized changes in neuronal recordings in humans has been established in several ways. Only a few studies have directly compared changes in activity from the same sites in the same individual, using the same behavioral measure. More often the comparison has been between fMRI and electrophysiologic changes in populations recorded from the same functional anatomic system as defined by lesion effects; in a few studies those systems have been defined by fMRI changes such as the “default” network. The fMRI-electrophysiologic relationships have been evaluated empirically by colocalization of significant changes, and by quantitative analyses, often multiple linear regression. There is some evidence that the fMRI-electrophysiology relationships differ in different cortical areas, particularly primary motor and sensory cortices compared to association cortex, but also within areas of association cortex. Although crucial for interpretation of fMRI changes as reflecting neural activity in human cortex, controversy remains as to these relationships. Supported by: Dutch Technology Foundation and University of Utrecht Grant UGT7685, ERC-Advanced grant 320708 (NR) and NIH grant NS065186 (JO)

Functional magnetic resonance imaging (fMRI) with the blood oxygen dependent (BOLD) signal has become a major tool to investigate human brain function. The goal of many of these studies is to establish the location and nature of the neuronal events that generate human cognitive processes. However, the BOLD signal that is imaged does not measure neuronal events, but rather hemodynamics. In many of those studies there is an implicit assumption that this signal has an invariant relation to activity of neurons, indeed reflecting their firing rate, an assumption often subsumed in titles of these papers such as “Neural mechanisms … ”. In the first decade of fMRI studies this implicit assumption was little challenged. However, a decade ago, Heeger and Ress (2002) raised it, asserting that establishing the relationship between neuronal activity and the fMRI signal “has emerged as one of the most important areas in neuroscience.” The resolution of it was considered essential to interpreting fMRI findings.

It is well-established that multiple hemodynamic processes contribute to the BOLD response, in addition to neurophysiological events and several sources of artifacts. Hemodynamic processes include oxygenation level (notably levels of deoxyhemoglobin), blood volume, and blood flow (Kim and Ogawa, 2012), all of which respond to changes in neuronal metabolic demand (Attwell et al., 2010). The relationship between BOLD response parameters such as onset time, amplitude, duration, and post-stimulus undershoot on the one hand, and neuronal activity expressed in terms of firing rate, bandwidth-limited local field potentials (LFPs) and evoked potentials on the other, is still a matter of intense research (Logothetis, 2008). Much of the research on “neurovascular coupling” has been conducted with animals (Logothetis, 2008), but recent opportunities with neurosurgical patients have led to studies comparing BOLD and neuronal activity, to elucidate their relationship.

To date, simultaneous comparison between the two modalities has only been possible in animals (Logothetis et al., 2001; Niessing et al., 2005). The simultaneous imaging and neuronal recording in visual cortex of anesthetized monkey (Logothetis et al., 2001) is of particular interest. That study indicated that the BOLD signal was most closely related to changes in LFPs, usually considered to reflect the afferent inputs to neurons, and less closely to the firing rate of action potentials which reflects the output of neurons. Whether these findings can be extrapolated to human brain is not clear, and studies with human subjects are needed to further our understanding of the exact nature of neurovascular coupling. Here we review a subset of human studies that have addressed this issue. One of the major goals of fMRI studies is identifying the specific brain region(s) associated with a cognitive process. Neuronal activity that is to be compared to this should be collected with comparable spatial resolution (Hermes et al., 2012). For this reason, we have excluded studies where the standard scalp electroencephalogram (EEG) was the measures of neuronal activity, as the EEG has relatively poor spatial resolution, due to the smoothing properties of scalp and skull. What we have included are those human studies that have recorded neuronal activity with a spatial resolution comparable to fMRI, intracranial recordings of electrocorticogram (ECoG) from subdural or intraparenchymal electrodes and LFPs and single neuron activity from microelectrodes.

These intracranial recordings are performed in a clinical setting. This imposes additional limitations. Perhaps the most serious is the nature of the patient population in whom these procedures are indicated. Essentially all these studies have been done on patients with intractable epilepsy. Whether findings in this population can be generalized to other populations is unknown, even though most studies avoid recording from cortex that has been related to the patient's epilepsy by the presence of interictal discharges, structural lesions or evidence of involvement in seizure onset. However, Bettus et al. (2011) compared BOLD connectivity measures in epileptic and non-epileptic cortex in these patients and found differences including inferring effects of epileptic cortex on the “normal” non-epileptic areas. They suggested that there were widespread alterations in neurovascular coupling in these patients. The presence of widespread hypometabolism in lateral temporal cortex well away from the epileptogenic zone was previously shown with fluorodeyoxyglucose positron emission scans (Engel et al., 1982). A second limitation of the human intracranial studies is that the extent of brain sampled in the recordings is restricted to those clinically relevant for that patient. In practice this often means that ECoG recording through subdural grids or multiple depth electrodes (stereoencephalography) cover wide areas of usually only one hemisphere, while LFP and single neuron recording comes from a much more restricted site, though sometimes in both hemispheres such as both medial temporal lobes including both hippocampi.

At present it is not technically possible or safe to perform simultaneous fMRI and intracranial recording in humans. There is one study (Ritter et al., 2008) that attempted to overcome this by inferring mass synchronized multiunit neuronal activity from very high frequency (600 Hz) scalp EEG, which can be recorded during fMRI, a technique which is also applicable to “normal” subjects, but required averaging a remarkably large number of task trials (over 4000), limiting its application. Moreover, as mentioned earlier, direct comparison requires measurement at the same resolution, a requirement not met by EEG. Finally, while there are a relatively large number of reports on ECoG-fMRI relationships from multiple centers, with some replication of findings, at present microelectrode-fMRI relationships have been reported by only two centers, the University of Washington (Ojemann et al., 2010) and University of California Los Angeles (Mukamel et al., 2005; Nir et al., 2007, 2008; Ekstrom et al., 2009). Each used different techniques and sampled different brain regions, with the only replication within the same center.

Apart from the complex mechanisms underlying the BOLD response, the electrophysiological measures are also far from straightforward. Single neuron activity and LFPs from intracortical electrodes measure from very small populations that do not necessarily represent activity of the larger population that shares a particular (sub)function. Hence a correlation with a much larger fMRI voxel is unlikely to yield a strong correlation (Logothetis, 2008). On the other hand an ECoG electrode collects signal from approximately half a million neurons but little is known of what type of neurons (presumably predominantly the large pyramidal cells). With the currently preferred spectral analyses it is becoming clear that many fluctuations are superimposed within neuronal populations, and that they appear to be driven by multiple slow oscillations (0–roughly 30 Hz) which may originate from deeper brain structures or from the cortical surface (Buzsáki and Wang, 2012). However, the meaning of the various frequencies and fluctuations is not clear yet, and it is even unclear whether oscillations are at play in higher frequency ranges (Engel et al., 2001) or whether the faster fluctuations merely reflect increased local communication (expressed as broadband gamma power changes) (Miller et al., 2009) (see also Buzsáki and Wang, 2012).

Additionally, comparison of BOLD with electrocortical recordings even in the same subjects is not always straightforward. For one, there is no adequate method for identifying the exact location of surface electrodes, for matching BOLD signal and neural activity. Due to a brainshift following leakage of CSF the cortical surface deforms, causing inaccuracies in matching CT images with electrode positions to presurgical MRI (Hermes et al., 2010). Electrodes can further shift during closing of the dura, making implant photographs inaccurate. One approach would be to smooth the fMRI images and/or interpolate between electrodes, but this does not solve a fundamental issue, namely that electrodes measure only from parenchyma immediately underneath the contact surface (i.e., 2.3 mm for ECoG). Activity can be quite different from tissue immediately adjacent to an electrode (Freeman et al., 2000; Slutzky et al., 2010), hence a mismatch of a few mm would already impact on the comparison of signals. Second, BOLD changes are observed not only near capillaries but also in draining veins (Roberts et al., 2007) and often in upstream vessels due to inflow effects (Gao and Liu, 2012). Unless special measures are taken to minimize those artifacts (Neggers et al., 2008), BOLD foci will be shifted some distance along the draining vessels. Thirdly, the ECoG signals are strongly affected by the method of signal referencing. Most groups use common averaging, but this can cause removal of relevant global signal fluctuations. Alternatively one may use electrodes that face the skull, or are positioned on top of another grid. These different methods yield different results. Despite all these concerns, comparing BOLD to same-resolution neural signals is likely to shed some light on neurovascular coupling.

The technique and findings of the studies reviewed here are presented in Tables 1–3. In those tables, the term LFP refers only to those recorded through microelectrodes, with those recorded from the more widely spaced subdural or depth electrodes referred to as ECoG, as the former are thought to represent activity within 250 microns of the electrode (Katzner et al., 2009), while the latter average over an area of 2.3 mm square or greater for the type of electrode arrays used in the studies reported here. This ECoG spatial sensitivity is still much better than scalp EEG. Most fMRI studies have sought changes occurring with specific tasks. Those studies are in Tables 1–2. They are further subdivided into those task based change studies where the fMRI and neural activity measures were obtained in the same subject (Table 1) from those where the fMRI and neural activity measures were explicitly compared, but derived from different subjects (Table 2). In all intracranial studies, although the same or very similar tasks are used in all subjects, the technical limitation that requires obtaining the fMRI and neural activity measures at different times, separated by days or longer, already introduces a substantial element of variability. When analyzed in the standard way, group fMRI findings have high reproducibility between repeat fMRIs but individual subject findings do not (Raemaekers et al., 2007). In that study individual subject reproducibility was thought to be related to differences in global signal to noise ratios. A recent report suggests that individual subject reproducibility can be substantially increased by analyzing the scans as relative activation maps normalized as a percentage of local excitation (Voyvodic, 2012). Alternative methods also reduce, but not eliminate, test-retest variability (Bennett and Miller, 2010; Birn, 2012). Obtaining fMRI and electrophysiologic data in different subjects likely introduces even more variability, particularly since electrical stimulation mapping of cortical sites essential for several human cognitive processes in this same patient population, including several language and recent verbal memory measures, has demonstrated substantial individual variability in their location (Ojemann and Dodrill, 1985; Ojemann, 1989; Ojemann et al., 1989). In recent years there has been much interest in the large scale networks identified by correlated spontaneous rest activity in fMRI. Table 3 presents studies of the neural activity correlates of these networks, again divided into those studies where the network investigated was identified on fMRI in the same subjects, and the one where the fMRI data was from different subjects than the neural activity.

**Table 1:** **Task based fMRI-neural activity comparisons obtained in same subjects**.

**Article**	**Cortex sampled^a^**	**Neural activity measure^b^**	**Task^c^**	**Comparison^d^**	**Findings^d^**	**Comment**
Puce et al., 1995	Unilateral sensori-motor	ECoG EP (4)	Touch, air pulse, hand squeeze, and median nerve stimulation	Anatomic visual colocalization. Comparison with unstimulated hemisphere	Colocalization even when lesion displaced sensory cortex	
Puce et al., 1997	Ventral OT	ECoG EP N200 (2)	Unfamiliar faces, vs. letter string object controls	Anatomic visual colocalization of significant changes	Colocalization fusiform gyrus; fMRI more extensive	
Lachaux et al., 2007	Posterior lateral T, inferior F,P	ECoG depths (3)	Semantic judgment on readable words vs. unreadable fonts control	fMRI activation within 10 or 15 mm of prolonged 40–150 Hz increase	Within 15 mm, 80% colocalized, compared to 47% for lower ECoG frequencies	
Ekstrom et al., 2009	Medial T, hippocampus	ME depths (6)	Navigating virtual environment vs. direction of arrow control	Correlation BOLD and LFP frequency bands 1–180 Hz, unit firing	Correlation only in para-hippocampal gyrus, positive only for LFP 4–8 Hz (*r* = 0.73); None for hippocampus, units	Further evidence of different relationships in different cortical areas
Ojemann et al., 2010	L,R lateral T	ME (9)	Silent word pair association learning vs. silent word reading, fixation controls	Colocalization to same 3 × 3 × 7 mm ROI of significant changes in BOLD, LFP 8–300 Hz power, unit firing	BOLD activation colocalized with increased LFP 50–250 Hz power, not to units. No colocalization to BOLD decreases	
Hermes et al., 2012	F,P,O	ECoG (8)	Visually cued finger movement vs. rest	Correlations within 8 mm of BOLD, ECoG 5–13, 13–30, 65–95 Hz power	Sensorimotor cortex: BOLD increase colocalized, positively correlated to 65–95 Hz, accounting for 46% BOLD variance	Considering entire area of covered cortex, negative 5–30 Hz correlation explains additional 13% of BOLD variance
Khursheed et al., 2011	FT, little P,O	ECoG (6)	Sternberg working memory delay period vs. fixation control	Correlation with 4–8, 30–200 Hz power	30–200 Hz correlation positive, 4–8 negative near BOLD increases; 4–8 Hz increases distant	
Conner et al., 2011	Left F>T>P>O	ECoG (11)	Visually cued noun and verb generation vs. scrambled control; Silent fMRI, Overt ECoG	Correlation BOLD-ECoG power in 7 frequency bands, 0.5–300 HZ for electrodes pooled by lobe, gyrus	Overall 13–30 Hz negative (*r* = −0.39), 60–120 positive correlation (*r* = 0.21). Differed by lobe, gyrus, not task (see text)	Considering lobe explains additional 6% of BOLD variance. Note that fMRI and ECoG tasks differ
Harvey et al., 2013	Left visual cortex (V1) and intraparietal sulcus (IPS)	ECoG (1)	Bars at different orientations, vs. mean luminance	ECoG power 1–125 Hz in vs. out of V1 population receptive field estimated from fMRI	V1: 3–120 Hz increase greater in, except 9–12 Hz greater out, when rest of 3–120 does not change. IPS: 3–25 Hz decrease vs. baseline	V1 9–12 Hz increase outside receptive field represents inhibition. fMRI shows as negative BOLD signal

Notes for row 1:

a *F, frontal; T, temporal; P, parietal; O, occipital; L, left; R, right*.

b *ECoG, electrocorticogram, unless indicated from subdural grids. EP, evoked potentials from ECoG. ME, microelectrodes, with local field potentials (LFP) and single neuron (units) action potentials. “depth” indicates recordings from depth electrodes. Number of subjects indicated in parentheses*.

c *vs., verses. Except as noted fMRI and neural activity task essentially the same*.

d *Units, single neurons; ROI, Region of interest*.

**Table 2:** **Task based fMRI-neural activity comparisons with fMRI and neural activity measures obtained in different subjects with the same task(s)**.

**Article**	**Cortex sampled^a^**	**Neural activity measure^b^**	**Task^c^**	**Comparison**	**Findings**	**Comment**
Huettel et al., 2004; Engell et al., 2012	TO	ECoG visual EP (9)	Visual checkerboard of varying durations	Changes in magnitude, form of EP vs. BOLD with varying durations at different sites	Inconsistent BOLD-EP relation: BOLD monotonic, non-linear increase with duration in O, inferiorT; EP: O sustained, inferior T phasic. No duration or power changes	Attempt at direct comparison to findings of Logothetis et al. (2001) in monkey. Concluded that EP-BOLD relation differed across brain regions. See text for recent reanalysis of non-phase-locked ECoG power
Mukamel et al., 2005	Auditory	ME depth (2)	Viewing movie	Correlation unit firing, LFP power 1–130 Hz convoluted with standard hemodynamic response function, and BOLD	Linear correlation positive with unit firing, (*r* = 0.39, 0.5) LFP 40–130 Hz, (*r* = 0.39, 0.46) negative 5–15 Hz. Unit activations colocalize with fMRI	Often cited as indicating unit firing rate related to BOLD signal magnitude. Unit and LFP 40–130 Hz activity confounded
Meltzer et al., 2008	FTPO including midline bilateral	ECoG (14)	Sternberg working memory, comparing varying load	Visual colocalization of fMRI, LFP power significant changes, pooled by lobe	30–50 Hz up with increased load all O sites. 5–13: up med F, down O, lat F. BOLD little over-lap except increase colocalized L precentral only	
Nir et al., 2007	Auditory bilateral	ME depth (3)	Viewing movie	Convoluted unit firing, LFP 40–130 Hz to hemodynamic response function. Correlations during viewing	Unit firing correlated with BOLD (*r* = 0.70) only when correlations between unit firing of nearby neurons present (*r* = 0.66). 40–130 Hz correlated with BOLD *r* = 0.62	BOLD correlation with unit firing only when unit firing inter-correlated
Privman et al., 2007	O, posterior lateral T	ECoG (7)	Viewing movie, object categorization faces, houses, tools	Convoluted ECoG 8–13, 13–30, 30–70 HZ to hemodynamic response function. Correlations during viewing.	60% of electrodes with significant 30–70 Hz correlated (*r* = 0.34) with BOLD in O, lateral T auditory cortex category selectivity of best ECoG electrodes > that of best BOLD	No data on selective category co-localization of ECoG and BOLD
McDonald et al., 2010	L inferior F, ventral TO, ST. Regions selected those with BOLD change	ECoG (6)	Primed vs. unprimed words	Randomization test of colocalization of significant ECoG 70–190 Hz power change at sites with significant BOLD change	Significant in all subjects at ventral OT, in only part for other areas. Concluded “tightly coupled”	No data on sites without fMRI change. Also compared to scalp magneto-encephalo-gram

Notes for row 1:

a *F, frontal; T, temporal; P, parietal; O, occipital; L, left; R, right*.

b *ECoG, electrocorticogram, unless indicated from subdural grids. EP, evoked potentials from ECoG. ME, microelectrodes, with local field potentials (LFP) and single neuron (units) action potentials. “depth” indicates recordings from depth electrodes. Number of subjects indicated in parentheses*.

c *vs., verses. Except as noted fMRI and neural activity task essentially the same*.

**Table 3:** **Large scale network fMRI-neural activity comparisons**.

**Article**	**Cortex sampled^a^**	**Neural activity measure^b^**	**Task**	**Comparison**	**Findings**	**Comment**
He et al., 2008	Sensory-motor network	ECoG (5)	Rest	Correlation within, between BOLD and ECoG bands, <0.5–200 Hz, at ROIs in, out of network during awake, rapid eye movement (REM), Slow wave sleep (SWS)	BOLD-ECoG <0.5–4 Hz positively correlated in network, not out. ROIs in correlated, not out BOLD-ECoG correlation in independent of arousal state. 50–100 Hz positively correlated BOLD during awake, REM not SWS	This and the next paper establish 50–110 Hz correlated activity on top of ultra slow correlated oscillations in these networks. Suggest 50–100 Hz related to conscious experiences
Ko et al., 2011	Default network: medial FP, Lateral FPT	ECoG (4)	Rest	Independent components of <0.1 Hz in spectral coherence across 1–120 Hz	BOLD power peak 0.015 Hz in network, not out. Spectral coherence 65–110 Hz, peaks at 0.015 Hz	
Bettus et al., 2011	Correlated networks T,P,O, medial F, hippocampus, thalamus	ECoG depths (5) Patients with temporal lobe epilepsy	Rest	Epileptic vs. non-epileptic regions interictal non-linear cross correlations ECoG-BOLD	BOLD connectivity: non-epileptic > epileptic; ECoG connectivity: epileptic > non-epileptic, all frequencies. Directionality in both: epileptic to non-epielptic	Epileptic-non-epileptic region differences vary BOLD vs. ECoG. Suggest widespread neurovascular coupling alterations in epileptic region
Nir et al., 2008	Auditory bilateral	ME depth (3) ECoG (2)	Rest, pure tone, awake, asleep	L-R correlation at <0.1, 0.1–1, >1 Hz.	Interhemispheric correlations in <0.1 Hz, 40–100 Hz ECoG power and unit firing. Greater with sleep. ECoG finding only in electrodes in auditory sensory network, not out	Extends ultra low frequency correlations in network to unit firing. Note that fMRI, ME and ECoG data from different subjects

*In first three publications, network defined by fMRI correlation in same subjects. In last paper, network location defined anatomically with neural activity and fMRI data obtained in different subjects*.

Notes for row 1:

a *F, frontal; T, temporal; P, parietal; O, occipital; L, left; R, right*.

b *ECoG, electrocorticogram, unless indicated from subdural grids. EP, evoked potentials from ECoG. ME, microelectrodes, with local field potentials (LFP) and single neuron (units) action potentials. “depth” indicates recordings from depth electrodes. Number of subjects indicated in parentheses*.

The studies of Tables 1 and 2 investigating task based changes utilize many different behavioral paradigms. Most of them compare neural activity and the BOLD signal during a defined behavioral state to that with a “control” condition, but several look for the similarity of changes with manipulation of one parameter within a behavioral condition [e.g., varying stimulus duration (Huettel et al., 2004) or load in a memory task (Meltzer et al., 2008) or between two anatomic areas in or out of a population visual receptive field (Harvey et al., 2013), or epileptic or non-epileptic cortex (Bettus et al., 2011)]. Moreover, the techniques used to establish BOLD-neural activity relationships fall into several categories. One group of studies examined anatomic colocalization of changes above a statistical threshold. That colocalization was established phenomenologically by visually matching (Puce et al., 1995; Huettel et al., 2004; Meltzer et al., 2008) or by a statistical comparison (Puce et al., 1997; Lachaux et al., 2007; McDonald et al., 2010; Ojemann et al., 2010). These studies address the practical question of what electrophysiologic change is likely to be present when a significant fMRI change is present, but neither analysis establishes a quantitative relation between BOLD and the neural activity. The remaining studies examine correlations between the magnitudes of the BOLD and neural signals, providing a quantitative relationship. In several studies, the neural signal was convoluted with a hemodynamic response function prior to correlation with the BOLD signal (Mukamel et al., 2005; Nir et al., 2007; Privman et al., 2007). Most correlation studies have evaluated the BOLD neural activity relationship statistically within a general multiple linear regression model. Given that different tasks are expected to elicit different localization of BOLD changes, the BOLD-electrophysiology coupling for different tasks might also differ. Few studies provide direct evidence on this. Conner et al. (2011) found no difference in ECoG correlative coupling for visually cued object naming or verb generation and Engell et al. (2012) found similar correlative coupling for different visual checkerboard stimulus durations, regardless of the presence or absence of a modulating effect of that parameter on BOLD and ECoG.

What can we conclude from these studies? Despite the differing sites sampled, tasks used and techniques for establishing BOLD-neural activity relationships, there are several. Most studies that have examined task based fMRI-neural activity relations in primary cortical areas, motor, somatosensory, auditory and visual, have reported a relation between increased BOLD signal and increased ECoG or LFP power in the “gamma” 30–130 Hz range (Tables 1 and 2). Single neuron firing has a similar relationship but only when there is correlated firing of nearby neurons. Then, single neuron firing has a quantitative linear relationship with BOLD activation. This is similar to the BOLD-neural activity relationship in monkey visual cortex recorded by Logothetis et al. (2001). However, in the Logothetis et al. study the relationship was to the lower range of “gamma” frequencies. One human study also found the occipital lobe relationship to this lower “gamma” range (Privman et al., 2007). Another human study that tried to directly replicate the Logothetis findings reported a similar positive relationship between the sustained evoked potentials and BOLD increases in peri-calcarine cortex but no consistent BOLD relationship to ECoG power, and no consistent relationship between BOLD and EPs as well as ECoG power in adjacent fusiform gyrus (Huettel et al., 2004). A recent re-analysis of the same data for non-phase-locked changes in high frequency power found a good correspondence between broadband gamma (30–100 Hz) and BOLD in both areas (Engell et al., 2012).

Increases in power in lower frequencies of ECoG-LFP also have a correlation with BOLD in these primary cortices but this relationship is negative; that increased power may be associated with either a negative BOLD signal (Harvey et al., 2013) or a smaller but still significant increase in the BOLD signal (Hermes et al., 2012). This relationship is less robust and somewhat independent of the relationship to “gamma” increases. The exact “low” frequency range has not been established, 9–12 Hz (Harvey et al., 2013), 5–15 Hz (Mukamel et al., 2005), 5–30 Hz (Hermes et al., 2012). Since there is increasing evidence that low frequencies are associated with suppression of cortical activity (e.g., Haegens et al., 2012; Miller et al., 2012; Harvey et al., 2013), tending to anticorrelate in amplitude with broadband gamma power (e.g., Hermes et al., 2012; Miller et al., 2012), it may be that there are no specific frequencies that drive the BOLD response.

The situation in association cortex is even less clear, but there too, despite the diversity of sites and tasks, most studies recording from neocortex have identified some positive correlation or colocalization with ECoG or LFP “gamma” frequencies, from 30–250 Hz. A few have also reported a negative correlation with increased power at lower frequencies, again in different ranges, 13–30 Hz (Conner et al., 2011) or 4–8 Hz (Khursheed et al., 2011). A relationship of BOLD to single neuron firing rates has not been established for association cortex. This may be due to the low firing rates of neurons there and the infrequent occurrence of correlated firing between nearby neurons (Ojemann et al., 2002).

Several studies suggest that the BOLD-neural activity relationships differ in different regions of cortex, even for the same task. Huettel et al. (2004) reported differences in the relationship between BOLD and ECoG evoked responses in peri-calcarine or fusiform cortex, though when analyzed by non-phase-locked ECoG power, these differences were not evident (Engell et al., 2012). In recordings from a large number of electrodes in wide areas of the left hemisphere during a language task, Conner et al. (2011) compared the BOLD-ECoG coupling when recordings were pooled by lobe to that of all areas, a positive correlation at 60–120 Hz and a negative one at 13–30 Hz. Significant differences from that average pattern of coupling were found for all lobes. They also compared the differences in BOLD-ECoG coupling for electrodes pooled by the relation to sites identified as critical for language based on electrical stimulation mapping (Ojemann et al., 1989) to those that were not. Critical language sites in superior temporal gyrus had greater coupling for 120–240 Hz, those in middle temporal gyrus less for 30–120 Hz, and in posterior inferior frontal gyrus (Broca's area), more for 30–240 Hz. fMRI activation has not shown sufficient specificity to these crucial language sites in individual patients to be a substitute for electrical stimulation mapping (Giussani et al., 2010 for review). In addition to the differences between neocortical areas discussed above, Ekstrom et al. (2009) found no significant coupling between BOLD and LFP power or single neuron firing in hippocampus, and the only relationship in the allocortex of the parahippocampal gyrus a positive correlation to 4–8 Hz LFP power. In those structures there was little relation between LFP “gamma” (or “theta”) power and single neuron firing (Ekstrom et al., 2007).

How much such regional differences reflect different degrees of engagement of different areas of cortex by a particular task, or inherent differences in the neurovascular coupling of different areas, or differences in their neuronal populations in their LFP power responsiveness is unknown (Ekstrom, 2010). Some insight into this issue comes from a study of the ability of LFPs in different frequency ranges to predict within 1 ms the timing of firing of individual neurons recorded through the same microelectrode, in recordings from human lateral temporal cortex (Zanos et al., 2012). Many of the neurons in which such a relationship could be established divided into two separate populations, those with timing of discharges predicted by 8–14 Hz LFP, and those by 80–150 Hz. Many of the recordings analyzed in that study were performed during the word association learning paradigm used in the Ojemann et al. (2010) study of BOLD-LFP relationships. Neurons with firing predicted by 80–150 Hz significantly increased activity with the learning paradigm, those with 8–14 Hz, to the word reading control. Experimental animal modeling of LFPs analyzed this way indicates that the 80–150 Hz activity represents input from neighboring neural circuits, while low frequencies originate from distant neuronal populations. In the Ojemann et al. (2010) study BOLD increased signal during the learning paradigm, compared to the control, colocalized with 50–250 Hz LFP power increases, suggesting that the BOLD signal reflects local synaptic input.

The large scale networks identified by rest activity in the fMRI that correlates across wide areas colocalize with low to ultra low frequency ECoG oscillations, less than 0.5–4 Hz in one study, less than 0.1 in two others, with a peak at 0.015 Hz in one (Table 3). This relationship has been identified in several large scale networks, including sensory-motor and the “default” network. Riding on these very slow oscillations are 40–110 Hz oscillations, which in the study of Ko et al. (2011) were most prominent at 0.015 Hz frequency of the slow oscillations. That single neuron firing rates correlate with the very slow oscillations is suggested in the one study where this was examined, where however, single neuron, ECoG and fMRI data were collected from different patients.

The above findings establish broadband “gamma” (30–250 Hz) ECoG-LFP power as a major feature of the neural activity coupled to the task based BOLD metabolic signal in humans in neocortex, independent of the specific task, thus providing a partial answer to Heeger and Ress challenge. However, it is also clear that the coupling may not be so simple as the invariant linear relation to neuron firing rates assumed in the early days of fMRI. Many aspects remain to be established, among them the role of decreases in power of lower ECoG-LFP frequencies, the extent and causes of the spatial variability in the coupling to the ECoG-LFP, the conditions under which the BOLD signal does reflect single neuron firing, and if so the nature of the relation, and the role of the ultra slow frequency oscillations of the large scale networks in modulating neural activity.

## Conflict of interest statement

The authors declare that the research was conducted in the absence of any commercial or financial relationships that could be construed as a potential conflict of interest.
